# Percutaneous nephrostomy vs. double-J stenting for emergency decompression in recurrent ipsilateral stone-related infectious obstruction

**DOI:** 10.3389/fmed.2026.1910507

**Published:** 2026-08-05

**Authors:** Wei Hu, Jing-jing Lou, Ze-peng Wu, Xiang Hu, Lidiao Li, Yu-han He

**Affiliations:** 1 Department of Intensive Care Unit, Yongkang First People's Hospital Affiliated to Hangzhou Medical College, Jinhua, China; 2 Department of Urology, Yongkang First People's Hospital Affiliated to Hangzhou Medical College, Jinhua, China

**Keywords:** double-J stenting, emergency decompression, obstructive urinary tract infection, percutaneous nephrostomy, urinary tract calculi

## Abstract

**Purpose:**

To compare percutaneous nephrostomy (PCN) and double-J (DJ) stenting as emergency decompression modalities in patients with recurrent ipsilateral stone-related infectious obstruction with systemic inflammatory response who subsequently underwent retrograde intrarenal surgery (RIRS) after infection control.

**Methods:**

This retrospective cohort study enrolled 69 patients (PCN, *n* = 35; DJ, *n* = 34) who underwent emergency decompression followed by retrograde intrarenal surgery (RIRS). Emergency decompression and perioperative RIRS outcomes were compared.

**Results:**

Baseline demographic and clinical characteristics were comparable between the groups. The drainage procedure time was longer in the PCN group than in the DJ group (42.53 ± 11.34 vs. 32.69 ± 10.72 min; MD, 9.84 min; 95% CI, 4.54 to 15.14; *P* < 0.001). However, PCN was associated with a shorter time to the first recorded body temperature below 37.3 °C after decompression (2.94 ± 1.26 vs. 4.36 ± 1.58 h; MD, −1.42 h; 95% CI, −2.11 to −0.73; *P* < 0.001) and a shorter length of stay during the decompression admission (3.26 ± 1.13 vs. 4.13 ± 1.29 days; MD, −0.87 days; 95% CI, −1.45 to −0.29; *P* = 0.004). During subsequent RIRS, UAS use was less frequent in the PCN group than in the DJ group (42.9% vs. 88.2%; RR, 0.49; 95% CI, 0.33 to 0.73; *P* < 0.001). The recorded RIRS operative time was shorter in the PCN group (50.35 ± 10.69 vs. 74.91 ± 14.35 min; MD, −24.56 min; 95% CI, −30.67 to −18.45; *P* < 0.001), as was postoperative hospital stay (2.84 ± 0.93 vs. 3.45 ± 1.28 days; MD, −0.61 days; 95% CI, −1.15 to −0.07; *P* = 0.028). Stone-free rates were comparable between the groups (91.4% vs. 88.2%; RR, 1.04; 95% CI, 0.88 to 1.22; *P* = 0.710).

**Conclusion:**

In this retrospective cohort of patients with recurrent ipsilateral stone-related infectious obstruction who achieved infection control and subsequently underwent RIRS, PCN was associated with a shorter time to the first recorded body temperature below 37.3 °C, a shorter length of stay during the decompression admission, a shorter recorded RIRS operative time, and a shorter postoperative hospital stay than DJ stenting, while stone-free rates were comparable. Because of the retrospective and non-randomized design, these findings should be interpreted as associations rather than causal effects.

## Introduction

Upper urinary tract calculi are a common urological condition and their incidence and recurrence rates have risen in recent years, placing a growing burden on patients' quality of life and on healthcare resources (1, 2). Clinically, stones may cause flank pain, hematuria, and hydronephrosis; once obstruction develops, however, impaired urinary drainage markedly increases the risk of urinary tract infection (3, 4). In particular, urinary tract infection secondary to obstruction may rapidly progress from a localized infection to systemic inflammation and, in severe cases, infection-associated organ dysfunction or septic shock (4–6).

Infectious upper urinary tract obstruction is a urological emergency because persistent obstruction and bacterial infection may lead to renal dysfunction, septic shock, multiple-organ dysfunction, and death (5, 6). Early recognition and timely intervention are therefore essential once stone-induced obstruction is complicated by infection (3, 5, 7). Current management emphasizes early fluid resuscitation, empirical broad-spectrum antibiotic therapy, and prompt urinary decompression once infectious obstruction is confirmed, all aimed at achieving source control (3, 5, 7). In clinical practice, ureteral stent insertion (double-J stent, DJ) and percutaneous nephrostomy (PCN) are the two most widely used emergency drainage modalities; both relieve obstruction and restore drainage within a relatively short period of time, thereby reducing intrarenal pelvic pressure (3, 8–10).

To date, most studies comparing PCN and DJ stenting have focused on acute obstructive infection caused by urinary calculi in general populations, whereas evidence specifically addressing recurrent ipsilateral stone-related infectious obstruction remains limited (11–19). Several studies have evaluated emergency drainage and subsequent ureteroscopic or retrograde intrarenal surgical outcomes after obstructive infection, but whether the decompression modality affects both acute recovery and later RIRS-related outcomes in patients with a documented separate ipsilateral recurrent episode remains unclear (17–19). In the present study, a recurrent ipsilateral episode was defined clinically as a separate ipsilateral hospitalization for stone-related infectious obstruction occurring after clinical resolution of the previous infectious episode. This definition was used to identify a clinically specific cohort rather than to imply that recurrent episodes are necessarily pathophysiologically different from initial episodes. Evidence regarding emergency decompression and subsequent definitive stone treatment in this recurrent population remains limited.

Accordingly, this study aimed to compare PCN and DJ stenting as emergency decompression modalities in patients with recurrent ipsilateral stone-related infectious obstruction with systemic inflammatory response who subsequently underwent RIRS after infection control.

## Methods and patients

This single-center retrospective cohort study included all consecutive patients who met the predefined inclusion and exclusion criteria and were admitted to the Yongkang First People's Hospital Affiliated to Hangzhou Medical College, between January 2020 and January 2026. The study enrolled patients presenting with a second ipsilateral episode of stone-related infectious obstruction accompanied by systemic inflammatory response caused by unilateral ureteropelvic junction or ureteral calculi. The present analysis included only patients who achieved infection control after emergency decompression and subsequently underwent RIRS. All patients underwent emergency urinary decompression during hospitalization, either by DJ or PCN, followed by retrograde intrarenal surgery (RIRS) as definitive treatment after infection was controlled. Patients were divided into the PCN group and the DJ group according to the decompression modality used during the second hospitalization. This study was approved by the Ethics Committee of Yongkang First People's Hospital Affiliated to Hangzhou Medical College (Approval No. 2026-LW-026), which waived the requirement for written informed consent because of the retrospective study design. In this study, a “second ipsilateral episode” was defined as a separate hospitalization for ipsilateral stone-related infectious obstruction accompanied by systemic inflammatory response after clinical resolution of the previous infectious episode. To avoid misclassifying persistent infection from the first episode as a second episode, only patients with documented clinical resolution of the previous infectious episode and a clearly separate subsequent hospitalization were included. The interval between the first and second emergency decompression procedures was defined as the time from emergency urinary decompression during the first ipsilateral hospitalization to emergency urinary decompression during the second ipsilateral hospitalization. This interval was not interpreted as the precise time to stone recurrence. All patients diagnosed with ureteropelvic junction or ureteral calculi were confirmed by non-contrast CT. Hydronephrosis was classified as mild, moderate, or severe according to imaging findings. Mild hydronephrosis was defined as dilatation of the renal pelvis without significant calyceal dilatation; moderate hydronephrosis as dilatation of the renal pelvis and calyces without obvious renal parenchymal thinning; and severe hydronephrosis as marked pelvicalyceal dilatation accompanied by renal parenchymal thinning.

Stone-related infectious obstruction with systemic inflammatory response was defined as imaging-confirmed unilateral upper urinary tract obstruction caused by calculi, together with confirmed or clinically suspected urinary tract infection and fulfillment of at least two systemic inflammatory response syndrome criteria. The SIRS criteria included: body temperature >38 °C or < 36 °C; heart rate >90 beats/min; respiratory rate >20 breaths/min or arterial partial pressure of carbon dioxide < 32 mmHg; and white blood cell count >12 × 10^9^/L or < 4 × 10^9^/L, or an immature granulocyte proportion >10%. The qSOFA score was collected as a baseline indicator of clinical severity but was not used as a diagnostic criterion for sepsis. Because the complete variables required for retrospective SOFA calculation were not consistently available, the study population was not classified as having Sepsis-3-defined sepsis.

The inclusion criteria were as follows: age ≥ 18 years; previous hospitalization for ipsilateral stone-related infectious obstruction accompanied by systemic inflammatory response; documented resolution of the previous infection; current admission for a second ipsilateral stone-related infectious obstructive episode accompanied by systemic inflammatory response; imaging confirmation of unilateral ureteropelvic junction or ureteral calculi and obstruction; emergency decompression by PCN or DJ stenting; subsequent RIRS after infection control; and availability of complete clinical data. Exclusion criteria included bilateral obstruction or bilateral stones; urinary tract obstruction not caused by stones; anatomical abnormalities of the urinary tract; pregnancy; concurrent other severe infections; subsequent definitive treatment by PCNL or another non-RIRS modality; failure to undergo subsequent RIRS; missing key data; or inability to clearly distinguish the first from the second episode.

All patients underwent routine laboratory testing, urine culture, and imaging evaluation upon emergency admission. Empirical intravenous broad-spectrum antibiotics were initiated immediately after the diagnosis of stone-related infectious obstruction. The initial regimen was selected according to the patient's clinical condition, renal function, allergy history, local antimicrobial practice, and suspected pathogen profile. Blood and urine cultures were obtained before or at the time of antibiotic administration whenever feasible. After culture and antimicrobial susceptibility results became available, treatment was adjusted accordingly, including escalation for resistant organisms and de-escalation to a narrower-spectrum agent when clinically appropriate. The same antibiotic-management protocol was applied to both groups. The operating room was contacted simultaneously to arrange urgent urinary decompression. The choice of emergency decompression was not randomized but was made by the attending urologist according to the patient's clinical condition, anesthesia tolerance, stone location, degree of hydronephrosis, technical feasibility, and patient preference. PCN was generally selected when retrograde drainage was considered difficult or when local anesthesia was preferred, whereas DJ stenting was selected when retrograde access was considered feasible. In the PCN group, percutaneous nephrostomy was performed under real-time ultrasound guidance using a disposable percutaneous nephrostomy dilation and drainage kit (model CDK16BFD; YiGaoMed, Zhejiang YiGao Medical Technology Co., Ltd., Zhejiang, China). Patients were placed in the prone or lateral position. After local anesthesia, the target calyx was punctured with an 18-gauge, 20-cm puncture needle. After successful entry into the collecting system, urine or purulent fluid was aspirated as much as possible and sent for culture. A 0.032-inch guidewire was then advanced into the renal collecting system, followed by tract dilation using the dilator supplied in the kit. Finally, a 16-Fr nephrostomy drainage catheter was inserted over the guidewire. Correct catheter position was confirmed by ultrasound, and the catheter was connected to external drainage. In the DJ group, ureteral stenting was performed in the lithotomy position under local or general anesthesia. Under cystoscopic or ureteroscopic guidance, a guidewire was advanced retrogradely across the obstructed segment into the renal pelvis. A 5-Fr/26-cm hydrophilic-coated double-J ureteral stent (model PUS5026EFA; YiGaoMed, Zhejiang YiGao Medical Technology Co., Ltd., Zhejiang, China) was then inserted over the guidewire. Successful stent placement was confirmed by visualization of the distal coil in the bladder and ultrasound confirmation of the proximal end in the renal pelvis.

Before definitive RIRS, all patients were re-evaluated after infection control. RIRS was performed only when body temperature had normalized and stabilized, the general condition had improved, and preoperative blood tests showed normal white blood cell counts and procalcitonin levels, with no evidence of active infection. RIRS was performed under general anesthesia with the patient in the lithotomy position. For patients initially decompressed with a DJ stent, the stent was first removed cystoscopically or ureteroscopically. A zebra guidewire was advanced into the kidney through the ureteroscope. Because of the retrospective design, UAS placement was not governed by a prospectively standardized protocol. A 12/14-Fr UAS was placed intraoperatively according to ureteral accessibility, technical feasibility, stone characteristics, the anticipated need for repeated ureteroscope passage, and surgeon preference. RIRS was then performed over the guidewire, and stones were fragmented using a 200-μm holmium:YAG laser. In the PCN group, the nephrostomy tube was opened during the procedure to reduce intrarenal pelvic pressure. At the end of surgery, a double-J stent was placed in all patients. All patients returned for follow-up 4 weeks later for stent removal and assessment of residual stone fragments. Postoperative fever was defined as a body temperature >38 °C occurring within 48 h after RIRS. Stone-free status was defined as complete absence of stones on follow-up at 4 weeks postoperatively, assessed by non-contrast CT.

Data collected primarily included patient baseline characteristics, outcomes related to emergency decompression, and outcomes related to subsequent RIRS. Baseline data included age, sex, body mass index (BMI), stone characteristics, comorbidities, degree of hydronephrosis, admission temperature, quick Sequential Organ Failure Assessment (qSOFA) score, and urine culture results. Emergency decompression-related outcomes included the admission-to-decompression interval, drainage procedure time, time to the first recorded body temperature below 37.3 °C, length of stay during the decompression admission, ICU admission, and the 5-point single-item global QoL score assessed at discharge. Time to the first recorded body temperature below 37.3 °C was defined as the interval from completion of emergency urinary decompression to the first recorded body temperature below 37.3 °C. Body temperature was routinely measured every 30 min during hospitalization. Quality of life was assessed at discharge using a single-item global patient-reported rating on a 5-point scale. Patients were asked to rate their overall perceived quality of life as follows: 1, very poor; 2, poor; 3, neither poor nor good; 4, good; and 5, very good. Higher scores indicated better perceived quality of life. RIRS-related outcomes included the decompression-to-RIRS interval, use of a ureteral access sheath (UAS), stone-free rate, operative time, postoperative fever within 48 h after RIRS, and postoperative hospital stay. Because this was a retrospective exploratory study, no primary outcome had been prospectively prespecified. For clarity, time to the first recorded body temperature below 37.3 °C after decompression was designated as the principal clinical outcome in the revised manuscript. The remaining emergency decompression and RIRS-related variables were considered secondary or exploratory outcomes.

All statistical analyses were performed using SPSS 26.0. Continuous variables were expressed as mean ± standard deviation, with comparisons between groups performed using the independent samples *t*-test; categorical variables are expressed as number (percentage), with comparisons between groups performed using the chi-square test or Fisher's exact test. The 5-point single-item global QoL score was treated as an ordinal variable, expressed as median and interquartile range, and compared using the Mann–Whitney U test. All tests were two-tailed, with *P* < 0.05 considered statistically significant. Effect estimates for continuous outcomes were expressed as mean differences (MDs) between the PCN and DJ groups with 95% confidence intervals (CIs). Effect estimates for categorical outcomes were expressed as risk ratios (RRs) comparing PCN with DJ, with 95% CIs.

## Results

During the study period, 497 patients were screened. Of these, 428 were excluded, mainly because they presented with a first ipsilateral episode of stone-related infectious obstruction accompanied by systemic inflammatory response (*n* = 396), underwent emergency decompression but did not subsequently proceed to RIRS (*n* = 17), or did not meet other eligibility criteria (*n* = 15). The remaining 69 patients were included in the final analysis, including 35 patients in the PCN group and 34 in the DJ stenting group. Baseline demographic and clinical characteristics are presented in Table 1. There were no statistically significant differences between the PCN and DJ groups in sex, age, BMI, stone size, number of stones, laterality, stone location, hypertension, diabetes, smoking history, degree of hydronephrosis, admission temperature, the proportion of patients with a qSOFA score ≥ 2, or urine culture positivity (all *P* > 0.05). The median interval between the first and second emergency decompression procedures was 25 months (IQR, 13–37) in the PCN group and 27 months (IQR, 14–40) in the DJ group, with no statistically significant between-group difference (*P* = 0.361).

**Table 1:** Baseline demographics and clinical characteristics of patients.

Variable	PCN (*n* = 35)	DJ (*n* = 34)	*p* value
Sex, *n*			0.733
Male	10	11	
Female	25	23	
Age (years)	57.34 ± 10.48	55.47 ± 11.53	0.484
BMI (kg/m^2^)	23.29 ± 4.34	24.17 ± 5.18	0.448
Stone size (mm)	8.38 ± 2.16	8.72 ± 2.37	0.536
Stone			0.496
Single	22	24	
Multiple	13	10	
Laterality, *n*			0.548
Left	19	16	
Right	16	18	
Stone location			0.894
Ureteropelvic junction	4	6	
Proximal ureter	17	16	
Middle ureter	10	9	
Distal ureter	4	3	
Hypertension, *n*			0.930
No	12	12	
Yes	23	22	
Diabetes, *n*			0.687
No	14	12	
Yes	21	22	
Smoker, *n*			0.729
No	21	19	
Yes	14	15	
Hydronephrosis, *n*			0.818
Mild	12	14	
Moderate	13	12	
Severe	10	8	
Temperature ( °C)	39.37 ± 0.91	39.47 ± 1.16	0.692
qSOFA ≥2, *n*	12	14	0.555
Urine culture			0.548
Positive	16	18	
Negative	19	16	
Interval between first and second ipsilateral episodes, months	25 (13–37)	27 (14–40)	0.361

BMI, body mass index; DJ, double-J; PCN, percutaneous nephrostomy; qSOFA, quick Sequential Organ Failure Assessment.

Clinical outcomes during the emergency decompression phase are presented in Table 2. The admission-to-decompression interval did not differ significantly between the PCN and DJ groups (83.27 ± 19.59 vs. 80.48 ± 17.61 min, *P* = 0.483). The drainage procedure time was longer in the PCN group than in the DJ group (42.53 ± 11.34 vs. 32.69 ± 10.72 min; MD, 9.84 min; 95% CI, 4.54 to 15.14; *P* < 0.001). In contrast, the PCN group had a shorter time to the first recorded body temperature below 37.3 °C after decompression (2.94 ± 1.26 vs. 4.36 ± 1.58 h; MD, −1.42 h; 95% CI, −2.11 to −0.73; P < 0.001) and a shorter length of stay during the decompression admission (3.26 ± 1.13 vs. 4.13 ± 1.29 days; MD, −0.87 days; 95% CI, −1.45 to −0.29; *P* = 0.004). ICU admission occurred in 3 patients (8.6%) in the PCN group and 2 patients (5.9%) in the DJ group (RR, 1.46; 95% CI, 0.26 to 8.19; *P* = 1.000). The median 5-point single-item global QoL score at discharge was higher in the PCN group than in the DJ group [4 (IQR, 2–4) vs. 3 (IQR, 2–4), *P* = 0.004]. Perioperative outcomes during the subsequent RIRS phase are also presented in Table 2. The decompression-to-RIRS interval did not differ significantly between the PCN and DJ groups (23.17 ± 9.44 vs. 24.64 ± 10.16 days, *P* = 0.718). UAS use was less frequent in the PCN group than in the DJ group [15/35 (42.9%) vs. 30/34 (88.2%); RR, 0.49; 95% CI, 0.33 to 0.73; *P* < 0.001]. Stone-free rates were comparable between the groups [32/35 (91.4%) vs. 30/34 (88.2%); RR, 1.04; 95% CI, 0.88 to 1.22; *P* = 0.710]. The recorded RIRS operative time was shorter in the PCN group than in the DJ group (50.35 ± 10.69 vs. 74.91 ± 14.35 min; MD, −24.56 min; 95% CI, −30.67 to −18.45; *P* < 0.001). Postoperative fever within 48 h after RIRS occurred in 3 patients (8.6%) in the PCN group and 5 patients (14.7%) in the DJ group (RR, 0.58; 95% CI, 0.15 to 2.25; *P* = 0.477). Postoperative hospital stay after RIRS was shorter in the PCN group than in the DJ group (2.84 ± 0.93 vs. 3.45 ± 1.28 days; MD, −0.61 days; 95% CI, −1.15 to −0.07; *P* = 0.028).

**Table 2:** Comparison of clinical outcomes between two groups.

Outcome	PCN (*n* = 35)	DJ (*n* = 34)	Effect estimate (95% CI)	*p* value
**Admission-to-decompression interval, min**	83.27 ± 19.59	80.48 ± 17.61	MD 2.79 (−6.16 to 11.74)	0.483
Drainage				
Operative time, min	42.53 ± 11.34	32.69 ± 10.72	MD 9.84 (4.54 to 15.14)	< 0.001^**^
Time to the first recorded body temperature below 37.3 °C after decompression, hours	2.94 ± 1.26	4.36 ± 1.58	MD −1.42 (−2.11 to −0.73)	< 0.001^**^
Hospital stay, days	3.26 ± 1.13	4.13 ± 1.29	MD −0.87 (−1.45 to −0.29)	0.004^**^
ICU Admission, *n* (%)	3	2	RR 1.46 (0.26 to 8.19)	1.000
QoL score, median (IQR)	4 (2–4)	3 (2–4)	MD 0.85 (0.22 to 1.48)	0.004^**^
**Decompression-to-RIRS interval, day**	23.17 ± 9.44	24.64 ± 10.16	MD −1.47 (−6.19 to 3.25)	0.718
RIRS				
UAS, *n* (%)	15	30	RR 0.49 (0.33 to 0.73)	< 0.001^**^
Stone free rate, *n* (%)	32	30	RR 1.04 (0.88 to 1.22)	0.710
Operative time, min	50.35 ± 10.69	74.91 ± 14.35	MD −24.56 (−30.67 to −18.45)	< 0.001^**^
Postoperative fever within 48 h after RIRS, *n* (%)	3	5	RR 0.58 (0.15 to 2.25)	0.477
Hospital stays, days	2.84 ± 0.93	3.45 ± 1.28	MD −0.61 (−1.15 to −0.07)	0.028^*^

Data are presented as mean ± standard deviation, median (interquartile range), or *n* (%), as appropriate. MD, mean difference; RR, risk ratio. MDs were calculated as PCN minus DJ, and RRs as PCN relative to DJ. Effect estimates are presented with 95% confidence intervals. DJ, double-J; ICU, intensive care unit; PCN, percutaneous nephrostomy; QoL, quality of life; RIRS, retrograde intrarenal surgery; UAS, ureteral access sheath; MD, Mean Difference; RR, risk ratio. ^*^P < 0.05, ^**^P < 0.01.

## Discussion

In patients with upper urinary tract calculi, once a stone causes obstruction complicated by infection, urinary stasis, elevated intrarenal pelvic pressure, and an increasing bacterial burden can rapidly drive local infection toward systemic inflammation, which in severe cases may progress to septic shock, multiple organ dysfunction syndrome, or even death. The management of infectious obstruction therefore depends not only on antimicrobial therapy but, more importantly, on achieving effective urinary decompression as promptly as possible (3, 5, 7). However, most previous studies have evaluated emergency decompression in general populations with stone-related infectious obstruction, whereas evidence specifically addressing patients with a documented recurrent ipsilateral episode remains limited (11–19). Xu et al. (12) reported that PCN achieves more rapid defervescence in patients with urosepsis. Similarly, Ibrahim et al. (19) found that compared with DJ, the PCN group had shorter time to the first recorded body temperature below 37.3 °C after decompression, shorter time to white blood cell normalization, and shorter hospital stay, indicating faster recovery. These observations are consistent with our finding that the PCN group recovered more quickly. This finding is readily explained on mechanistic grounds: by establishing a direct external drainage pathway, PCN more rapidly reduces intrarenal pelvic pressure, clears infected urine, and reduces persistent stimulation of the obstructed system by inflammatory mediators. Stone-related urinary obstruction accompanied by infection and systemic inflammatory response represents a clinically serious condition requiring prompt urinary decompression. Previous urinary tract infection or stone history may be associated with an increased risk of sepsis or acute pyelonephritis in patients with ureteral calculi. However, current evidence does not demonstrate that a recurrent ipsilateral episode is necessarily more severe than an initial episode. Structural or mucosal changes after the previous episode were not systematically assessed in the present retrospective cohort. Therefore, the recurrent ipsilateral population should be regarded as a clinically defined cohort rather than as a distinct pathological entity. The present findings do not establish that recurrent episodes are inherently more difficult to control or require a different decompression strategy. Nonetheless, the literature has not reached a consensus on whether PCN has a definitive advantage across all patients with infectious obstruction. A retrospective cohort study by Anil et al. (14) concluded that both PCN and DJ are effective and safe decompression modalities; although complications were more frequent in the DJ group, these were predominantly mild. The 2024 meta-analysis by Moon et al. (15) further reported that, among patients with severe urinary tract infection complicated by obstructive urolithiasis, the two drainage modalities showed no consistent statistically significant differences in time to the first recorded body temperature below 37.3 °C after decompression, time to white blood cell normalization, hospital stay, or procedural success rate. Goldsmith et al. (13) in a 15-year retrospective study, likewise found both modalities to be effective but observed that clinicians tended to favor PCN for emergency drainage in patients with larger stones and more severe disease. Retrospective analyses are therefore susceptible to confounding factors such as individual physician preference, underscoring the substantial inconsistency among current studies and the considerable influence of selection bias.

Another clinically relevant finding was the potential influence of the initial decompression modality on subsequent RIRS (17–19). Despite comparable stone-free rates, the PCN group had a shorter RIRS operative time and a shorter postoperative hospital stay. Several factors may account for this difference. First, patients in the DJ group required intraoperative removal of the indwelling stent before RIRS. Second, UAS placement was more frequent in the DJ group, which may have increased procedural complexity and operative time. Third, in the PCN group, the nephrostomy tube could be left open during RIRS to aid drainage and reduce intrarenal pelvic pressure. Opening the nephrostomy tube during RIRS may provide an additional drainage route and potentially reduce intrarenal pressure. However, intrarenal pressure was not directly measured in the present study. Pressure-related complications, fluid extravasation, and rare stone migration outside the collecting system have been reported after endoscopic stone surgery, underscoring the importance of appropriate postoperative clinical and imaging follow-up (20). The shorter recorded RIRS operative time in the PCN group should be interpreted cautiously. Therefore, the observed difference cannot be attributed solely to the initial decompression modality.

The findings of Ibrahim et al. (19) are consistent with ours: the PCN group had a shorter subsequent RIRS operative time and fewer postoperative fever events, whereas stone-free rates did not differ significantly. In contrast, Liao et al. (17), comparing flexible ureteroscopy outcomes after initial retrograde ureteral stenting vs. PCN in patients with prior urosepsis, found no significant differences in operative time, postoperative fever, postoperative hospital stay, or stone-free rate. Pietropaolo et al. (18) likewise reported that, in patients who had undergone emergency decompression for obstruction-complicated sepsis, subsequent elective ureteroscopy yielded generally favorable outcomes—a mean operative time of approximately 42 min and a stone-free rate of 97%—with no significant differences in operative time, complications, or stone-free rate between those who initially received PCN vs. retrograde stenting. Thus, whether the initial decompression modality affects the outcomes of subsequent RIRS or URS remains unresolved (17–19). With respect to patients' subjective experience, the 5-point single-item global QoL score assessed at discharge was higher in the PCN group than in the DJ group. Previous studies comparing PCN and ureteral stenting have also reported differences in patient-reported quality of life and treatment-related tolerance (21, 22). Indwelling ureteral stents may cause flank or abdominal discomfort, urinary frequency, urgency, and other lower urinary tract symptoms, whereas nephrostomy tubes may cause inconvenience related to external drainage (22, 23). However, the score used in the present study was a single-item global patient-reported measure rather than a validated multidimensional or drainage-specific QoL questionnaire. It may also have been influenced by residual symptoms, treatment-related discomfort, analgesic use, and patient expectations. Therefore, this finding should be interpreted as an exploratory patient-reported outcome rather than definitive evidence of a quality-of-life advantage of PCN.

This study has several limitations. First, it was a single-center retrospective study in which the choice of PCN or DJ stenting was not randomized; moreover, the emergency decompression procedures were not performed by a single surgeon, which may have introduced procedural and selection bias. Second, the inclusion criteria required patients to achieve infection control and subsequently undergo RIRS. Patients who did not proceed to definitive RIRS were therefore not represented, which may have introduced selection bias. Consequently, the findings apply only to patients who recovered sufficiently to undergo subsequent endoscopic stone treatment. Third, the QoL measure was a subjective global score rather than a validated stent- or nephrostomy-specific questionnaire. Fourth, patients were identified using evidence of stone-related infectious obstruction together with SIRS criteria (24). Complete data required for retrospective SOFA calculation were not consistently available; therefore, the cohort could not be reliably reclassified according to Sepsis-3. The present findings should consequently not be interpreted as applying specifically to all patients with Sepsis-3-defined sepsis.

## Conclusion

In this selected cohort of patients with recurrent ipsilateral stone-related infectious obstruction who achieved infection control and subsequently underwent RIRS, both PCN and DJ stenting achieved emergency urinary decompression. PCN was associated with a shorter time to the first recorded body temperature below 37.3 °C after decompression, a shorter decompression-related hospital stay, and shorter RIRS-related procedural parameters, while stone-free rates were comparable between the groups. However, because of the retrospective, single-center, and non-randomized design, as well as potential selection and procedural confounding, these findings should be interpreted as associations rather than causal effects. Further prospective studies are required to confirm these observations.

## Data Availability

The raw data supporting the conclusions of this article will be made available by the authors, without undue reservation.
